# A Novel Parasitoid of Marine Dinoflagellates, *Pararosarium dinoexitiosum* gen. et sp. nov. (Perkinsozoa, Alveolata), Showing Characteristic Beaded Sporocytes

**DOI:** 10.3389/fmicb.2021.748092

**Published:** 2021-11-29

**Authors:** Boo Seong Jeon, Myung Gil Park

**Affiliations:** LOHABE, Department of Oceanography, Chonnam National University, Gwangju, South Korea

**Keywords:** host range, parasitism, life cycle, phylogeny, ultrastructure

## Abstract

The phylum Perkinsozoa is known as an exclusively parasitic group within alveolates and is widely distributed in various aquatic environments from marine to freshwater environments. Nonetheless, their morphology, life cycle, the identity of the host, and physiological characteristics remain still poorly understood. During intensive sampling along the west coast of Korea in October and November 2017, a new parasitoid, which shares several characteristics with the extant families Perkinsidae and Parviluciferaceae, was discovered and three strains of the new parasitoid were successfully established in cultures. Cross-infection experiments showed that among the examined planktonic groups, only dinoflagellates were susceptible to the new parasitoid, with infections observed in species belonging to eight genera. Even though the new parasitoid shared many morphological and developmental characteristics with other Perkinsozoan parasites, it differed from them by its densely packed trophocyte structure without a large vacuole or hyaline material during the growth stage. These characteristics are common among Parviluciferaceae members. Furthermore, through palintomic extracellular sporogenesis, it produced characteristic interconnected sporocytes resembling a string of beads. Phylogenetic analyses based on the small subunit and large subunit ribosomal DNA sequences revealed that the new parasitoid was distantly related to the family Parviluciferaceae and was more closely related to the families Perkinsidae and Xcellidae. Morphological, ultrastructural, and molecular data on the new parasitoid raised the need to erect a new family, i.e., Pararosariidae, within the phylum Perkinsozoa with *Pararosarium dinoexitiosum* gen. et sp. nov. as the type species. The isolation and establishment in culture of the new parasitoid outside the family Parviluciferaceae in the present study would contribute to the better understanding of the diversity of Perkinsozoan parasites and provide useful material for comparisons to other parasite species in the further study.

## Introduction

The phylum Perkinsozoa is an exclusively parasitic group within alveolates, and it occupies the earliest branching phylogenetic position within the Dinozoa clade (Bachvaroff et al., 2014). The first described parasite belonging to this phylum was genus *Perkinsus*, which infects mollusks. This parasite was initially classified within the phylum Apicomplexa (Levine, 1978), being characterized by having an apical complex consisting of a conoid, rhoptries, micronemes, and polar rings. Ultrastructural studies on genus *Perkinsus* showed that the parasite had an incomplete conoid, thereby raising a question about its inclusion within the Apicomplexa which has a conoid (Perkins, 1988). Later, phylogenetic analyses of ribosomal genes showed that the *Perkinsus* parasite was closer to Dinoflagellata (Siddall et al., 1997). Subsequently, Norén et al. (1999) reported a new parasitoid (i.e., parasite which ultimately kills the host) of dinoflagellates, *Parvilucifera infectans*, having the similar ultrastructural features and close phylogenetic relationship with *Perkinsus* and newly erected the phylum Perkinsozoa to encompass the two genera, independent of Apicomplexa.

Among Perkinsozoan parasites inhabiting marine environments, to date, only three families have been described morphologically and taxonomically: Perkinsidae Levine, 1978, which comprises one genus infecting mollusks such as abalone, clams, oysters, and scallops (Lester and Davis, 1981; Dubremetz et al., 1998; Casas et al., 2004; Dungan and Reece, 2006; Moss et al., 2008); Parviluciferaceae Reñé et al., 2017a, which comprise four genera parasitizing dinoflagellates (Norén et al., 1999; Reñé et al., 2017a; Jeon and Park, 2019); and Xcellidae Freeman et al., 2017, which comprises three genera infecting various fish species (Freeman et al., 2017; Karlsbakk et al., 2021). Very recently, a new parasitoid of marine dinoflagellates, *Maranthos nigrum* Reñé et al., 2021, which is distantly related to the family Parviluciferaceae, was added to the phylum Perkinsozoa (Reñé et al., 2021). By contrast, all known Perkinsozoan parasites from freshwater environments remain partially characterized in terms of morphology or nuclear ribosomal DNA (rDNA) sequences. For example, no genetic information on *Rastrimonas subtilis* (Brugerolle) infecting the freshwater algae cryptophyte is available at present, but observations of its ultrastructure support that this parasite belongs to the phylum Perkinsozoa (Brugerolle, 2002, 2003). Sequences of the infectious agent of tadpoles in freshwater environments, which was possibly associated with amphibian mortality events, have also been reported (Davis et al., 2007). These sequences were clustered with numerous environmental sequences obtained from freshwater environments and formed a clade referred to as Novel Alveolate Group 01 (NAG01) (Chambouvet et al., 2015), whereas no detailed information on their morphology and life cycle is available. Recently, an intriguing internal association between a Perkinsozoan parasite and the colonial chlorophycean species *Sphaerocystis* was detected using tyramide signal amplication-fluorescent *in situ* hybridization (TSA-FISH) (Mangot et al., 2013; Jobard et al., 2020), but the parasite was not characterized morphologically.

Environmental molecular studies revealed that Perkinsozoan parasites are widely distributed in various aquatic environments from marine to freshwater environments, as well as in harsh conditions such as the abyssal sea floor, hydrothermal vents, sediment, and peat bog (López-García et al., 2003; Lepère et al., 2008; Behnke et al., 2010; Bråte et al., 2010; Scheckenbach et al., 2010; Lara et al., 2011; Mangot et al., 2011; Chambouvet et al., 2014). Owing to advances in molecular genetic techniques including next generation sequencing (NGS), the ubiquitous distribution and great genetic diversity of these taxa have been further unveiled over the past two decades (Mangot et al., 2009, 2013; Lepère et al., 2010; Jobard et al., 2020). Nonetheless, Perkinsozoa is still full of numerous environmental rDNA sequences without taxonomically-defined, with their morphology, life cycle, the identity of the host, and physiological characteristics remaining unknown. Therefore, conventional methods (e.g., infected single-cell isolation, cultivation, and microscopic observation) are indispensable to name those environmental rDNA sequences and to taxonomically expand species diversity within the Perkinsozoa group. Using these conventional methods on free-swimming stages (i.e., zoospores), however, may be considerably difficult, mostly because zoospores are generally very small in size and survival outside their hosts is limited (e.g., Alacid et al., 2015; Jeon and Park, 2019), thereby making difficult to search for and observe. This may also apply to Syndinian parasites, a sister group of the Perkinsozoa; they have also been widely detected in various marine environments (e.g., Guillou et al., 2008; De Vargas et al., 2015).

During intensive sampling along the west coast of Korea in October and November 2017, a new parasitoid, which shares several characteristics with the extant families Perkinsidae and Parviluciferaceae, was discovered and three strains of the new parasitoid were successfully established in cultures. Using the cultures, the morphology and development of this new parasitoid were systematically investigated through light and electron microscopy. Remarkably, its life cycle, especially the sporogenesis stage, differed from that of previously described Perkinsozan parasites. Morphological, ultrastructural, and molecular data on the new parasitoid raised the need to erect the new family Pararosariidae in the phylum Perkinsozoa with *Pararosarium dinoexitiosum* gen. et sp. nov. as the type species.

## Materials and Methods

### Parasitoid Detection, Isolation, and Cultivation

Concentrated seawater samples were taken using a 20-μm mesh plankton net through vertical towing from the bottom to the surface at the west coast of Korea, i.e., in Hongwon harbor (36°09′24″ N, 126°30′08″ E) in October 2017, and in Gyeokpo harbor (35°37′19″ N, 126°28′10″ E) in November 2017. In addition, mixed samples containing seawater and sediment from rocky tide pools at the Gyeokpo shoreline (35°37′24″ N, 126°28′02″ E) were collected in November 2018 using a hand shovel. All samples were pre-filtered using a 200-μm mesh to remove large zooplankton and potential predators, and the samples were then transported to the laboratory. Dinoflagellates identified in seawater samples included *Akashiwo sanguinea*, *Tripos furca*, *Dinophysis acuminata* complex, and *Gonyaulax* sp. in samples from Hongwon harbor, as well as *Akashiwo sanguinea*, *Tripos* spp., *Dinophysis* spp., *Gonyaulax* sp., *Oxyphysis* sp., and *Prorocentrum micans* in samples from Gyeokpo harbor. In the sample collected in the rocky tide pools, *Prorocentrum minimum* was the sole observed dinoflagellate, and it occurred at a very low density. Aliquots (2 mL) of each sample were distributed in wells of a 6-well plate (Corning Inc., Corning, New York, United States) containing 1 mL of the mixed dinoflagellates cultures (*Akashiwo sanguinea* strain As-LOHABE07, *Alexandrium pacificum* strain Ap-LOHABE05, and *Scrippsiella acuminata* strain Sa-LOHABE02) as potential hosts, followed by incubation in f/2 -Si medium (Guillard and Ryther, 1962) at 20°C under a 14:10 h light:dark cycle with cool-white fluorescent light providing 100 μmol photons m^–2^ s^–1^. For detection of parasitoids, all samples were examined daily using an inverted microscope (Axio Vert.A1, Carl Zeiss Inc., Hallbergmoos, Germany). Rounded body structures within the dinoflagellates cells were considered a sign of infection with a Perkinsozoan parasitoid (Norén et al., 1999; Figueroa et al., 2008; Reñé et al., 2017a,b; Jeon and Park, 2019, 2020; Alacid et al., 2020), and infection was detected solely in the dinoflagellate *Alexandrium* cells among the mixed dinoflagellates cultures added as potential hosts. Infected dinoflagellate cells were manually isolated using a drawn-glass micropipette under an inverted microscope, washed six times in syringe-filtered seawater (0.45 μm pore size filter; Advantec, Tokyo, Japan) from the samples, and were then individually transferred to a 96-well plate (Corning Inc.) containing 20 μL of exponentially growing *A. pacificum* strain Ap-LOHABE05 culture (Jeon and Park, 2019, 2020). Each isolate that showed successful infection was re-isolated thrice with the same procedure using single infected host cells to preclude contamination. The parasitoids were propagated by sequentially transferring aliquots of either infected *A*. *pacificum* cells or sporocytes to exponentially growing *A*. *pacificum* cultures using plant culture dishes (SPL Lifesciences, Gyeonggido, Korea) twice per week, followed by maintenance under the same growth conditions as described above for the hosts. Cultures of the three parasitoid strains were thus successfully established.

### Light Microscopy

For light microscopy observations of the life cycle and morphology of the different stages of the new parasitoid, exponentially growing *A*. *pacificum* cultures were inoculated in a 6-well plate with recently formed (<3 h) zoospores, which were harvested by gravity filtration through a 10 μm pore size membrane filter (Millipore, Cork, Ireland) from the parasitoid stock culture (Jeon and Park, 2020), and the culture was subsequently incubated under the same growth conditions as described above. Light microscopic images were captured at 200-, 630-, and 1,000-fold magnification using a digital cinema camera (Canon EOS C300 mark2; Canon Inc., Tokyo, Japan) coupled to an Axio Imager A2 microscope (Carl Zeiss Inc.) equipped with differential interference contrast optics and epifluorescence ability. To examine nuclear division, aliquots (100 μL) of inoculated cultures were fixed with 2% glutaraldehyde (final concentration) and were stained using 5X SYBR Gold (final concentration) (Molecular Probes, Eugene, Oregon United States) at 4°C for 1 h. Stained cultures were then photographed using epifluorescence microscope with blue light excitation (Filter Set 09; excitation BP 450–490, beam splitter FT 510, emission LP 515). Videos were recorded at 200- and 630-fold magnification using an Axio Imager A2 microscope equipped with a digital cinema camera to observe parasitoid development. For enumeration of the zoospores produced per late trophocyte, each late trophocyte was placed individually in a hemocytometer and was allowed to develop zoospores under humid conditions, and mature zoospores were counted subsequently (Jeon and Park, 2019). At this time, the diameter of the late trophocyte was also measured using an inverted microscope (Axio Vert.A1, Carl Zeiss Inc.) coupled to a full HD mini box camera (MediCAM-Z, Comart System, Korea).

### Electron Microscopy

For scanning electron microscopy (SEM) observations, late trophocytes were manually isolated from the parasitoid culture using a drawn-glass micropipette, were placed on rounded cover glasses (14 mm in diameter) with syringe-filtered seawater, and were allowed to develop zoospores under humid conditions. Each sample was fixed successively using 2% glutaraldehyde (final concentration) at 4°C for 24 h to attach the parasitoids to the cover glass. Fixed samples were placed in distilled water for 1 h and were dehydrated in a graded ethanol series (25, 50, 70, and 99%) for 12 min per step and were then rinsed thrice using absolute ethanol for 15 min. Samples were critical-point dried in liquid CO_2_ using a HCP-2 device (Hitachi, Tokyo, Japan). The rounded cover glasses were subsequently glued to SEM stubs using carbon tape, were sputter-coated with platinum, and were examined using a Hitachi HR-SEM (model SU-70; Hitachi) operated at 15 kV. For transmission electron microscopy (TEM) observations, aliquots of the parasitoid cultures collected at successive times after zoospores inoculation were fixed using 2.5% glutaraldehyde (final concentration) buffered with 0.1 M cacodylate buffer at pH 7.4 and were then stored at 4°C until processing. The fixed samples were processed at the Korea Basic Science Institute (KBSI, Ochang, Korea) following the procedures detailed by Jeon and Park (2019). Sections were examined using TEM at 120 kV (Technai G2 Spirit Twin; FEI, Hillsboro, OR, United States).

### Screening of Microorganisms for *Pararosarium dinoexitiosum* Infection

To assess the infection potential of the parasitoid, exponentially growing hosts belonging to several taxa (50 strains, 8 orders, and 20 genera) were examined. All host cultures used in this test came from LOHABE culture collection, Chonnam National University, Republic of Korea. The potential hosts contained several taxa: dinoflagellates (43 strains from 24 species), raphidophytes (2 species), Euglenophyta (1 species), Ciliate (1 species), and cryptophytes (3 species) (Table 1). Each host strain was inoculated at a concentration of 2 × 10^3^ cells mL^–1^ with recently formed (<3 h) zoospores at a concentration of 240 × 10^3^ cells mL^–1^ in a 24-well plate, and infections were conducted in triplicates at a final volume of 1 mL. This approach generated a zoospore:host ratio of 120:1. The inoculated cultures were incubated for 10 days under the same growth conditions as described above, and they were examined daily using an inverted microscope (Axio Vert.A1, Carl Zeiss Inc.). Detection of infection (i.e., trophocyte, empty trophocyte wall, and sporocyte) was confirmed until formation of new zoospores. When no infection was observed, the host strains were inoculated once more under the same conditions described above to confirm resistance to infection.

**TABLE 1:** Strains of dinoflagellates and other microorganisms infected with *Pararosarium dinoexitiosum*.

Class	Order	Genus	Species	Strain	Infected	Infected strains (%)	*n*
Dinophyceae	Gonyaulacales	*Alexandrium*	*affine*	Aa-LOHABE03	Yes		1
			*pacificum*	Ap-LOHABE01∼5	Yes	100	5
			*catenella*	Ac-LOHABE01∼4	Yes	100	4
		*Coolia*	*canariensis*	Cc-LOHABE01	No		1
			*monotis*	Cm-LOHABE01	Yes		1
		*Fragilidium*	*duplocampanaeforme*	Fd-LOHABE01	No		2
			*mexicanum*	Fm-LOHABE01	Yes		1
		*Ostreopsis*	sp. 1		Yes		1
		*Pyrophacus*	*steinii*	Pste-LOHABE01	Yes		1
	Gymnodiniales	*Akwashio*	*sanguinea*	As-LOHABE02∼7	No		6
		*Amphidinium*	cf. *trulla*	bdAT-LOHABE02	No		1
		*Grammatodinium*	*tongyeonginum*	Gt-LOHABE01	No		1
		*Gymnodinium*	*inusitatum*	Ginu-LOHABE01	No		1
		*Karenia*	*mikimotoi*	Km-LOHABE01	No		1
		*Levanderina*	*fissa*	Lf-LOHABE01	Yes	100	2
	Peridiniales	*Heterocapsa*	*triquetra*	Ht-LOHABE01∼3	No		3
			*Arctica*	Ha-LOHABE01∼2	No		2
			sp.	HtMH1	No		1
		*Scrippsiella*	*acuminata*	Sa-LOHABE01∼3	Yes	100	3
			*precaria*	Sp-LOHABE01	Yes		1
		*Thecadinium*	*kofoidii*	bdTK-LOHABE01	No		1
	Prorocentrales	*Prorocentrum*	*micans*	Pmic-LOHABE01	Yes		1
			*minimum*	Pmin-LOHABE01	No		1
			*triestinum*	Ptri-LOHABE01	No		1
Raphidophyceae	Chattonellales	*Fibrocapsa*	*japonica*	Fj-LOHABE01	No		1
		*Heterosigma*	*akashiwo*	Haka-LOHABE01	No		1
Euglenoidea	Euglenales	*Euglena*	sp.	Eugl-LOHABE01	No		1
Litostomatea	Cyclotrichiida	*Mesodinium*	*rubrum*	MR-LOHABE01	No		1
Cryptophyceae	Pyrenomonadales	*Chroomonas*	*mesostigmatica*	Cm-LOHABE01	No		1
			sp.	MhBe2	No		1
			sp.	DhBe2	No		1

*In case of > 1 strain, the percentage of infected strains is shown. n = the number of tested strains.*

### DNA Extraction, PCR Amplification, and Sequencing

Free-swimming zoospores were harvested from each of the parasitoid strains using gravity filtration through 10-μm pore size membrane filters (Millipore) to remove host cells, after which they were transferred to several new 1.5 mL tubes (Axygen Scientific, Union City, CA, United States) and pelleted by centrifugation (2,000 × g) at 4°C for 1.5 min. DNA was extracted from the pellet using a DNA Extraction Kit (Bioneer, Daejeon, Korea), and DNA extracts were stored at –20°C until processing. Polymerase chain reaction (PCR) amplifications of the small subunit (SSU) and the large subunit (LSU) ribosomal RNA (rRNA) genes, purification, and sequencing were performed as described previously (Jeon and Park, 2019). All produced sequences were deposited in GenBank (accession nos. MZ663823 and MZ668307 of strain PAdin-LOHABE01; MZ663824 and MZ668305 of strain PAdin-LOHABE02; MZ663830 and MZ668306 of strain PAdin-LOHABE03).

### Phylogenetic Analyses

The obtained sequences were aligned using the ContigExpress (vector NTI version 10.1, Invitrogenm NY, United States), and low quality regions were manually checked. The SSU rDNA sequences related to the sequences obtained in this study were identified through BLASTN similarity searches^1^. The obtained SSU rDNA sequences were primarily aligned with a total of 112 published sequences belonging to the family Parviluciferaceae, Perkinsidae, and Xcellidae, tadpole pathogens and environmental sequences recovered from the NCBI non-redundant (nr) database using MAFFT v7 (Katoh et al., 2019). SSU rDNA sequences belonging to three dinoflagellates (accession numbers KF885226, EU780638, and DQ779985), six belonging to Syndiniales group I (JN934987, JN934988, JN606065, MN388915, FJ440625, and AB264776), three belonging to Syndiniales group II (AF069516, AY775285, and HQ658161), and three belonging to Syndiniales group IV (DQ146404, DQ146406, and EF065717) served as the outgroup. This selection was further refined manually using MEGA7 (Kumar et al., 2016). Ambiguously aligned positions were removed, and final alignments of 1,524 nucleotide sites were selected. Maximum likelihood (ML) analysis was performed using IQ-TREE v1.6 (Nguyen et al., 2015) with the GTR + F + R5 model, determined as best fitting the data by ModelFinder (Kalyaanamoorthy et al., 2017), as implemented in IQ-TREE, and based on the Akaike information Criterion. To evaluate node supports, 300 non-parametric bootstrap trees were reconstructed using the same method. Bayesian analysis was performed using MrBayes v3.2.1 (Ronquist et al., 2012) with the GTR + I + G model running four simultaneous Markov chain Monte Carlo chains for 2,000,000 generations and sampling every 100 generations, following burn-in of 2,000 generations. Further, the combined SSU and LSU rDNA sequences were aligned using the same procedures as described above, and final alignments of 2,492 nucleotide sites were used. Phylogenetic relationships were determined as described above. The ML and Bayesian analysis were performed using GTR + F + R3 and GTR + I + G models, respectively.

## Results

### Life Cycle of *Pararosarium dinoexitiosum* and Morphology of Its Developmental Stages

**FIGURE 1:**
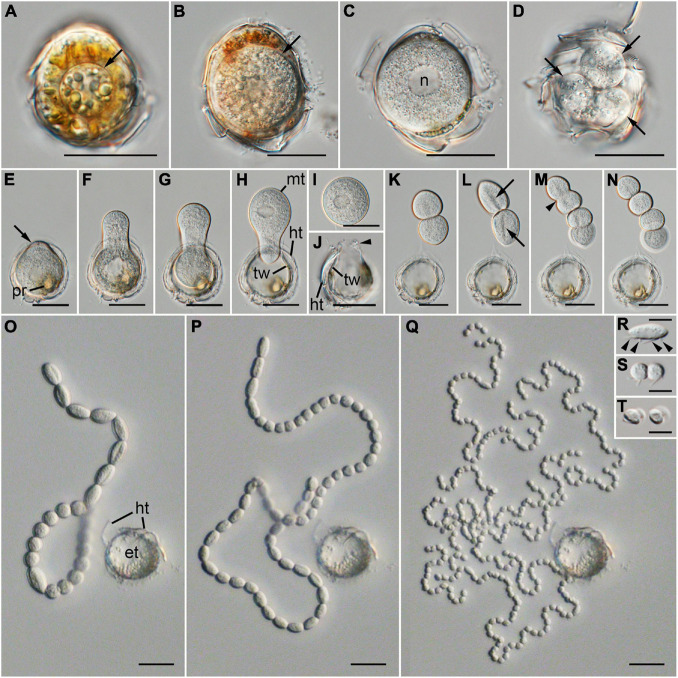
Differential interference contrast (DIC) images showing the life-cycle stages of *Pararosarium dinoexitiosum* gen. nov. et sp. nov. in the dinoflagellate host *Alexandrium pacificum*. **(A)** Early trophocyte (arrow) within host cytoplasm. **(B)** Growing trophocyte (arrow) consuming the host cytoplasmic contents. **(C)** Late trophocyte with a large nucleus (n). **(D)** Multiple infection showing three trophocytes (arrows) within a single host cell. **(E)** Wall of late trophocyte showing a bulge (arrow) with plastid remnants (pr) of host **(F–H)** Emergence of the mature trophocyte (mt) from the late trophocyte wall (tw). Theca remnants (ht) of host is also shown. **(I)** Complete emergence of a mature trophocyte. **(J)** Empty trophocyte with the mark of a mature trophocyte emerging (arrowhead), late trophocyte wall (tw), and theca remnants (ht) of host. **(K)** Two completely divided sporocytes. **(L)** Two sporocytes showing a long transversal groove (arrows) on the central surface. **(M)** Invagination (arrowhead) of the sporocytes during cytokinesis. **(N)** Four completely divided sporocytes. **(O–Q)** Progressive sporogenesis stages during the development of beaded sporocytes, resembling a rosary, with theca remnants (ht) of the host and empty trophocyte wall (et) of the parasitoid. **(R)** Sporocyte before the last division with four flagella (arrowheads). **(S)** Two completely divided immature zoospores with round body shape. **(T)** Sigmoid-shaped immature zoospores. Scale bars: **(A–Q)** = 20 μm, **(R–T)** = 5 μm.

**FIGURE 2:**
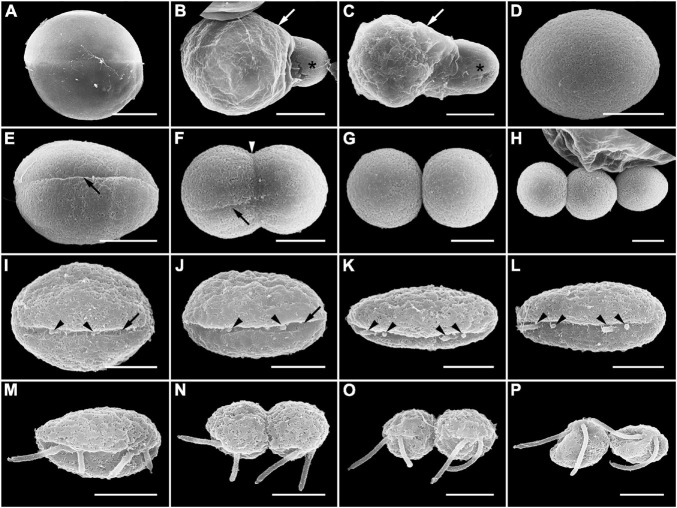
Scanning electron microscopy (SEM) images showing the late trophocyte and sporocyte during sporogenesis stages of *Pararosarium dinoexitiosum*. **(A)** Late trophocyte with smooth surface of the wall. **(B,C)** Mature trophocyte (asterisk) emerging from the trophocyte wall (arrow). **(D)** Complete emergence of mature trophocyte showing rounded body and smooth surface. **(E)** The initial stage of sporocyte division showing a long transversal groove (arrow) on the central surface of one side. **(F)** The middle stage of the sporocyte division showing an invagination (arrowhead) in the direction perpendicular to transversal groove (arrow). **(G)** Two completely divided sporocytes. **(H)** Three linked sporocytes. **(I–L)** Sporocyte before the last division stage showing a gradual change into an elongated body shape and development of the flagella (arrowheads) from the transversal groove (arrow) of the body. **(M)** Fully developed flagella of a sporocyte. **(N,O)** Last division of the sporocyte showing rounded immature zoospores with flagella. **(P)** Immature zoospores that are gradually changing into the sigmoid shape. Scale bars: **(A–C)** = 10 μm, **(D–H)** = 5 μm, **(I–P)** = 2 μm.

**FIGURE 3:**
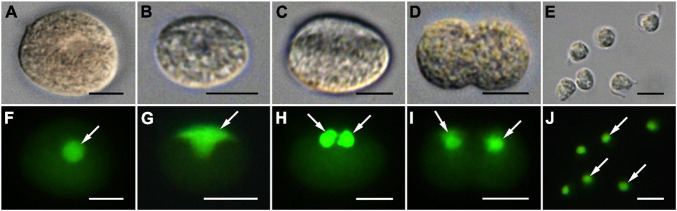
Differential interference contrast (DIC) **(A–E)** and SYBR gold-stained epifluorescence images **(F–J)** showing division of the nucleus during sporogenesis stages of *Pararosarium dinoexitiosum*. The images in each column show the same specimen. **(A,F)** Sporocyte with a SYBR gold-stained large and rounded nucleus (arrow). **(B,G)** Sporocyte with laterally positioned T-shape nucleus (arrow). **(C,H)** Sporocyte showing two completely divided nuclei (arrows). **(D,I)** Sporocyte showing two nuclei (arrows) separated from each other during cytokinesis. **(E,J)** Immature zoospore containing a small and rounded nucleus (arrows). All scale bars = 5 μm.

Infection was established when a zoospore actively penetrated the dinoflagellate host *A. pacificum* strain Ap-LOHABE05, during which the host lost its swimming ability and sank to the bottom of the culture dish. Presence of a round body in the host cytoplasm (Figure 1A) indicated infection, which was easily recognizable through light microscopy 24 h after zoospore penetration. The round body of the parasitoid, which is an intracellular feeding stage and is also referred to as trophocyte, continued to consume the host and increased in size until it occupied most of the host cell (Figure 1B). When the host cytoplasm was almost completely consumed 48 h after penetration, the late trophocyte showing a wall with a smooth surface (Figure 2A) had a large nucleus (9.17 ± 0.42 μm, *n* = 5; 29 ± 1.45 μm in late trophocyte diameter, *n* = 5) (Figure 1C). In the case of penetration of several zoospores, multiple infections were commonly observed within a single host *A. pacificum* cell, and their trophocytes were smaller than those observed after single infection (Figure 1D). When host cell density was low or no host cells were present in the culture dish, the late trophocyte occasionally remained as a resting dormancy stage until it was activated by an additional supply of healthy host cells. Once activated, it began to bulge out in one random direction (Figure 1E). Then, the mature trophocyte was successively released from the trophocyte wall into the surrounding water (Figures 1F–H, 2B,C). In the case of multiple infections, individual late trophocytes bulged out from its own trophocyte wall in the order of activation and the resultant mature trophocytes were released from the trophocyte wall into the environment through different holes. This series of release processes from bulging of a trophocyte wall to complete emergence of the mature trophocyte in single infection took approximately 45 min. After emergence of the mature trophocyte, the mark of emergence on the empty trophocyte wall could be easily observed using light microscopy (Figure 1J). The mature trophocyte that had just emerged showed a typically spherical shape with a smooth surface and was densely packed (Figures 1I, 2D). At this stage, the mature trophocyte sequentially transformed into a sporocyte, and it underwent palintomic sporogenesis extracellularly (Figures 1K–Q). During palintomic sporogenesis, karyokinesis began with a change in the laterally positioned nucleus from a round to a T-shape (Figures 3A,B,F,G). After nuclear fission, the newly produced nuclei began to move away from each other before completion of cytokinesis (Figures 3C,D,H,I). During these processes, a long transversal groove was observed on the central surface of one side of the sporocyte (Figures 1L, 2E). After karyokinesis, cytokinesis occurred via invagination in the direction perpendicular to the transversal groove of the binucleate sporocyte, yielding two daughter sporocytes (Figures 1M,N, 2F,G). This cycle of division processes was repeated 8–9 times over a period of approximately 3 h. These successive divisions yielded sporocytes that were linked together, resembling a string of beads, which was the most prominent morphological characteristic of the parasitoid (Figures 1O–Q, 2H). The sporocyte successively elongated before the last division, whereas the flagella began to sprout from the transversal groove of the body (Figures 2I–L). At this stage, the flagella were developed and the final sporocytes possessed four active flagella (Figures 1R, 2M). After development of the flagella, cytokinesis began and resultant two separate immature zoospores with a round body shape were produced (Figures 1S, 2N,O). Although immature zoospores wriggled using their flagella, the bodies of the zoospores progressively changed into a sigmoid shape (Figures 1T, 2P). At this stage, the zoospores showed a small and round nucleus (1.36 ± 0.03 μm, *n* = 3) (Figures 3E,J). When the zoospores were fully developed, they dispersed in the water column to infect a new host. Under the growth conditions described above with *A. pacificum* as a host, the life cycle of the new parasitoid *P. dinoexitiosum* from host cell penetration to maturation of newly produced zoospores took approximately 65 h (Supplementary Video 1). The number of newly produced zoospores depended on the biovolume of the late trophocyte. When single or multiple infections occurred in host *A. pacificum*, the late trophocyte had a diameter of 21.2–24 μm (corresponding to 5,008–7,235 μm^3^ in volume) and produced 46.7 ± 0.8 zoospores per 10^3^ μm^3^ late trophocyte volume (mean ± SE, *n* = 5).

### Zoospore

**FIGURE 4:**
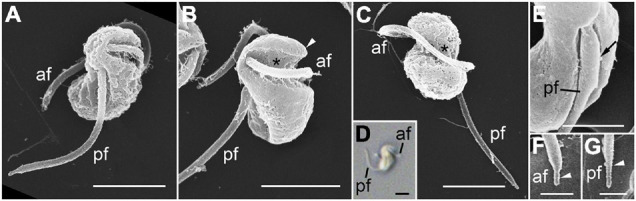
Scanning electron microscopy (SEM) **(A–C,E–G)** and differential interference contrast (DIC) **(D)** images of the zoospore of *Pararosarium dinoexitiosum*. **(A)** Lateral view of a zoospore showing the anterior flagellum (af) and the posterior flagellum (pf). **(B)** Ventral view of zoospore showing the rostrum (arrowhead), the anterior flagellum (af) with a groove (asterisk) of the cell, and the posterior flagellum (pf). **(C)** Dorsal view of zoospore showing the anterior flagellum (af) with a groove (asterisk) and the posterior flagellum (pf). **(D)** Zoospore showing sigmoid shape with the anterior flagellum (af) and the posterior flagellum (pf). **(E)** Posterior flagellum (pf) of zoospore showing the proximal paraxial swelling (arrow). **(F)** Detail of the anterior flagellum (af) showing short conical tip (arrowhead). **(G)** Detail of posterior flagellum (pf) showing distal shrunken end (arrowhead). Scale bars: **(A–D)** = 2 μm, **(E)** = 1 μm, **(F,G)** = 0.5 μm.

The zoospores were sigmoid-shaped in dorsal or ventral views and had a rostrum at the anterior body area (Figure 4). They were 2.97 ± 0.06 μm long and 1.65 ± 0.04 μm wide (*n* = 8, using SEM). The zoospores possessed two heteromorphic flagella, which were orthogonally inserted in proximity of each other on the anterior half of the body (Figure 4A). The hairy anterior flagellum, 8.22 ± 0.06 μm long (*n* = 3, using SEM), emerged transversely from the body, encircled the body in a groove of the cell (Figures 4A–D), and terminated in a short conical tip (Figure 4F). The acronematic posterior flagellum, 5.36 ± 0.11 μm long (*n* = 8, using SEM), ran longitudinally to the posterior end of the body (Figures 4A–D), and was characteristics of a proximal paraxial swelling and distal shrunken end (Figures 4E,G).

**FIGURE 5:**
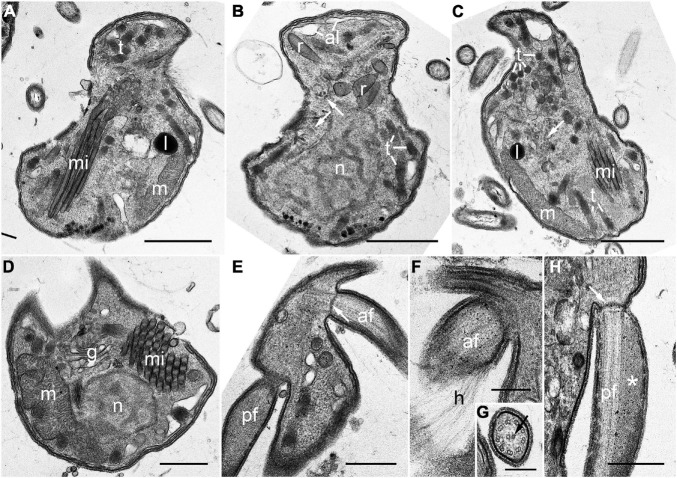
Transmission electron microscopy (TEM) images of the zoospore of *Pararosarium dinoexitiosum*. **(A)** Longitudinal section of a zoospore showing bipartite trichocysts (t), micronemes (mi), lipid globule (l), and a mitochondrion (m) with tubular cristae. **(B)** Longitudinal section of a zoospore showing alveoli (al), rhoptries (r), bipartite trichocysts (t), conoid-associated micronemes (arrows), and the nucleus (n) with condensed chromatin in a reticulated pattern. **(C)** Longitudinal section of a zoospore showing bipartite trichocysts (t), conoid-associated micronemes (arrow), micronemes (mi), the mitochondrion (m), and a lipid globule (l). **(D)** Transverse section of a zoospore showing a nucleus (n), micronemes (mi), the Golgi apparatus (g), and the mitochondrion (m). **(E)** Section showing the anterior flagellum (af) with transverse septum in the transition zone (arrow) and the posterior flagellum (pf). **(F)** Section showing the anterior flagellum (af) with hairs (h). **(G)** Detail of the axoneme of anterior flagellum showing a heteromorphic pair of central microtubules (arrow). **(H)** Longitudinal section of the posterior flagellum (pf) showing the proximal paraxial swelling (asterisk) and transverse septum in the transition zone (arrow). Scale bars: **(A–C)** = 1 μm, **(D–F,H)** = 0.5 μm, **(G)** = 0.25 μm.

Mature zoospores contained the following main components: alveoli, a Golgi body, lipid globules, a mitochondrion with tubular cristae, several bipartite trichocysts consisting of an electron-dense body (square in cross section) and a twisted filamentous head, and a rounded nucleus with condensed chromatin in a reticulated pattern (Figures 5A–D). Numerous micronemes were distributed over the central part of the cell from the posterior to the anterior end (Figures 5A–D). The apical complex structure comprised rhoptries, micronemes with bulbous ends, and conoid-associated micronemes (Figures 5B,C), however, a pseudo-conoid (open-conoid or incomplete conoid) was not observed in this study. The basal body of both anterior and posterior flagella did not contain a dense globule (Figure 5E). The hairy anterior flagellum showed an axoneme with a heteromorphic pair of central microtubules (Figures 5F,G), whereas the axoneme of the posterior flagellum was not heteromorphic and this flagellum showed the characteristic of paraxial swelling (i.e., a wing-like extension) (Figure 5H).

### Ultrastructure of Developmental Stages

**FIGURE 6:**
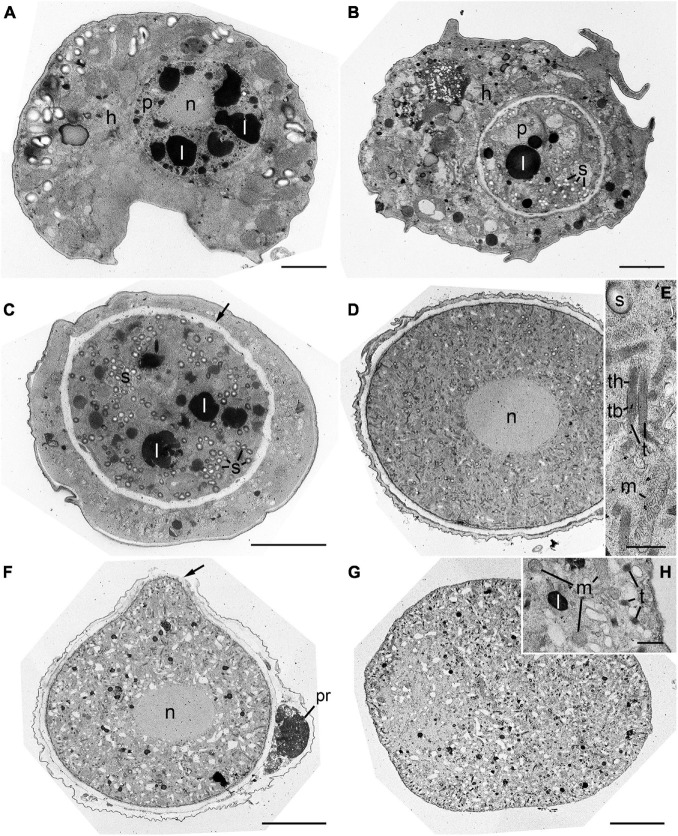
Transmission electron microscope (TEM) images during development stages of *Pararosarium dinoexitiosum*. **(A)** The initial stage of the infection showing the trophocyte of parasitoid (p) containing a nucleus (n) and lipid globules (l) within the host cytoplasm (h). **(B)** Early trophocyte (p) containing starch granules (s) and lipid globule (l) within host cytoplasm (h). **(C)** Middle to late trophocyte showing the distinct trophocyte wall (arrow), lipid globules (l), and starch granules (s). **(D)** Late trophocyte showing a large non-condensed nucleus (n) and densely packed structure. **(E)** Enlargement of the late trophocyte showing the bipartite trichocyst (t) consisting of a head (th) and body (tb), mitochondrion (m), and starch granule (s). **(F)** Late trophocyte showing a nucleus (n) and the bulge of a trophocyte wall (arrow) with plastid remnants (pr) of host. **(G)** Sporocyte showing compact structure. **(H)** Enlargement of the sporocyte showing the mitochondria (m), lipid globule (l), and bipartite trichocysts (t). Scale bars: **(A–D,F,G)** = 5 μm, **(E,H)** = 0.5 μm.

The early trophocyte was located in the host cytoplasm and was separated from the host by a parasitophorous vacuole membrane. At this stage, the trophocyte contained non-condensed nucleus and several lipid globules (Figure 6A). The early trophocyte gradually grew until it reached the size of the host while consuming its cytoplasmic contents. At this stage, numerous starch granules and lipid globules were observed, and the wall of the trophocyte was developing (Figures 6B,C). After the host cytoplasm was almost completely consumed, the late trophocyte showed a large-sized non-condensed nucleus at its center and contained numerous bipartite trichocysts consisting of head and body, mitochondria, and starch granules (Figures 6D,E). Subsequently, the wall of the fully developed trophocyte began to bulge out in one direction (Figure 6F), and the sporocyte emerging from the trophocyte wall showed a compact structure with several bipartite trichocysts with bodies square in cross section, mitochondria, and lipid globules (Figures 6G,H).

**FIGURE 7:**
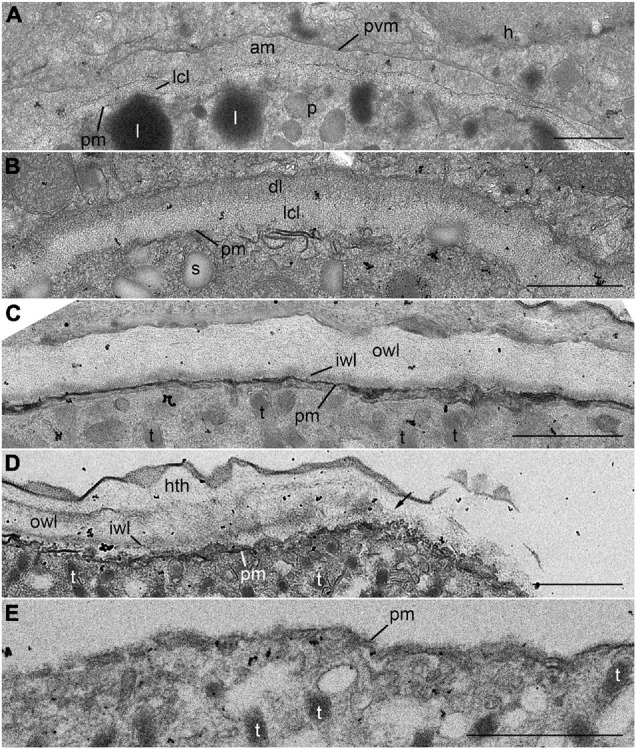
Transmission electron microscope (TEM) images of the trophocyte wall **(A–D)** and cell membrane of sporocyte **(E)** during the development stages of *Pararosarium dinoexitiosum*. **(A)** The wall of the initial trophocyte (p) comprising a parasitophorous vacuole membrane (pvm), amorphous material (am), less compact layer (lcl), and plasma membrane (pm) within host cytoplasm (h). Lipid globules (l) are also shown. **(B)** The wall of the early trophocyte showing electron-dense layer (dl) and less compact layer (lcl) with plasma membrane (pm). Starch granule (s) are also shown. **(C)** The fully developed wall of the trophocyte consisting of two layers: an outer wall layer (owl), an inner wall layer (iwl) with plasma membrane (pm). Bipartite trichocysts (t) are also shown. **(D)** The bulge of trophocyte wall showing the accompanied breaking (arrow) of outer wall layer (owl) and inner wall layer (iwl). Host theca (hth), plasma membrane, and bipartite trichocysts (t) are also shown. **(E)** Sporocyte showing the plasma membrane (pm) and bipartite trichocysts (t). All scale bars = 1 μm.

The trophocyte wall underwent several structural changes during the intracellular stage within host cell (Figure 7). At the initial stage of infection, the early trophocyte was clearly distinguishable from the host cytoplasm by an envelope consisting of three layers: an inner less compact layer, a layer of amorphous substance, and a parasitophorous vacuole membrane (Figure 7A). As the trophocyte grew by consuming host cytoplasm, the amorphous substance disappeared, and the trophocyte showed a wall consisting of two different layers: a less compact layer and an electron-dense layer (Figure 7B). At the middle to late trophocyte stage, the trophocyte wall was fully developed and comprised two distinct layers: an inner thin layer and a thicker less compact outer layer. The surface of the trophocyte wall was smooth without protrusion or invagination (Figure 7C). When the late trophocyte wall began to bulge out, the inner and outer layers were cut off by the emergence of the mature trophocyte (Figure 7D). At the sporogenesis stage, the sporocyte was enveloped in a plasma membrane and contained several bipartite trichocysts with organelles (Figure 7E).

### Screening of Microorganisms for *Pararosarium dinoexitiosum* Infection

No infection was observed in species belonging to Chattonellales, Euglenales, Cyclotrichiida, and Pyrenomonadales except for dinoflagellates (Table 1). Among the examined dinoflagellates, species belonging to eight genera (*Alexandrium*, *Coolia*, *Fragilidium*, *Ostreopsis*, *Pyrophacus*, *Levanderina*, *Scrippsiella*, and *Prorocentrum*) were susceptible to *P*. *dinoexitiosum*. No inter-strain variability in host response within a same species was observed. Species belonging to the order Gonyaulacales tended to be more susceptible than those from other orders.

### Phylogeny

SSU rDNA sequences of the three strains *P. dinoexitiosum* (length = 1,597–1,709 bp) had 99.9–100% similarity, with only one nucleotide difference of the strain PAdin-LOHABE02. The SSU rDNA sequences of *P*. *dinoexitiosum* showed the highest similarity (92.3%) with environmental sequence (AF530536) from hydrothermal Mid Atlantic Ridge. The LSU rDNA sequences obtained for the three strains *P. dinoexitiosum* (length = 1,417–1,478 bp) were 100% identical and showed highest similarities (88.7%) with those of both *Perkinsus andrewsi* (AY305327) and *Perkinsus atlanticus* (AF509333). The molecular phylogenetic tree inferred based on SSU rDNA sequences indicated that the new parasitoid in this study was a member of the phylum Perkinsozoa, with a low bootstrap value (ML = 72%) and a low posterior probability (0.7) (Figure 10). The Perkinsozoa group comprised two separate clades, one containing all Parviluciferaceae members, *M. nigrum*, which was placed at the base of Parviluciferaceae and formed a distinct long branch with low statistically support, and numerous marine and freshwater environmental sequences (43%/0.51) and the other containing the two families Perkinsidae and Xcellidae and numerous marine and freshwater environmental sequences including the NAG01 clade, which encompasses the sequence of the infectious agent of tadpoles (24%/0.42). The new family Pararosariidae containing the new species *P. dinoexitiosum* was distantly related to all members of the family Parviluciferaceae, however, it clustered with the families Perkinsidae and Xcellidae. The family Pararosariidae was placed at the base of the group comprising numerous marine and freshwater environmental sequences, including those of a pathogen of tadpoles with low statistical support (43%/0.58); it showed a sister relationship with the two families Perkinsidae and Xcellidae (86%/0.98).

The phylogenetic tree inferred based on combined SSU and LSU rDNA sequences also showed a similar topology to that inferred from SSU rDNA sequences (Supplementary Figure 1), although *Snorkelia* spp. (FJ424512, MF197552), Perkinsea sp. 2 (MT649884), *Salmoxcellia vastator* (MW743278-81), and tadpoles pathogen (EF675616) included only SSU rDNA sequences due to the lack of available LSU rDNA sequences. All Parviluciferaceae members formed a monophyletic clade with low supports (75%/1) and the sequences of *M. nigrum* was placed at the base of Parviluciferaceae. The three families Perkinsidae, Xcellidae, and Pararosariidae clustered together with low support (51%/0.96), and the new family Pararosariidae was a sister clade of the families Perkinsidae and Xcellidae (72%/0.99).

## Discussion

The development and application of molecular techniques helped reveal the large genetic diversity of the phylum Perkinsozoa over the past decade, however, knowledge of the morphology, life cycle, and biological and ecological characteristics of this group remain still poorly understood. In this context, the finding of the new parasitoid *Pararosarium dinoexitiosum* from this study, which mostly relied on conventional techniques such as isolation, cultivation, and microscopic observation, would mean more than a simple addition of one species to the Perkinsozoa group. First, the present study demonstrated the presence of a new parasitoid of dinoflagellates in the Perkinsozoa group at family level, which did not cluster within the family Parviluciferaceae. Second, observations of morphology and life cycle of the new parasitoid would provide insights into the biological character evolution in the Perkinsozoa group.

**FIGURE 8:**
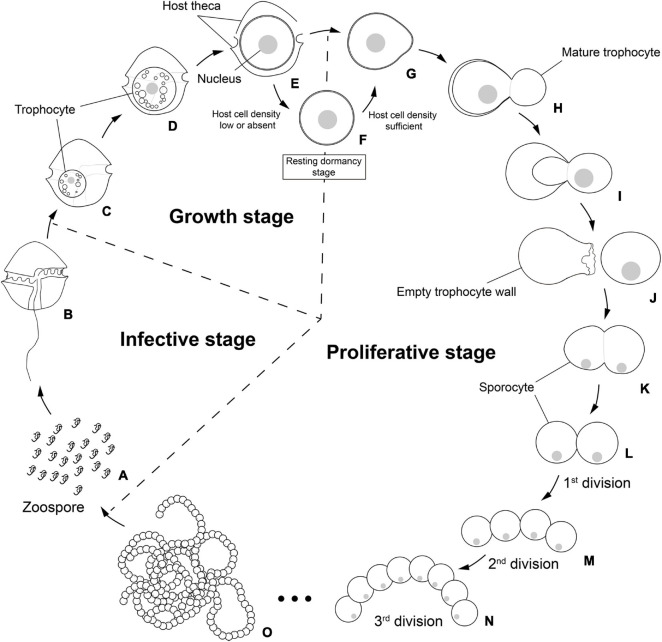
Schematic life cycle of the parasitoid *Pararosarium dinoexitiosum* infecting the dinoflagellate host *Alexandrium pacificum*. The life cycle of the parasitoid comprises three stages, an infective stage **(A,B)**, a growth stage **(C–E)**, a resting dormancy stage **(F)**, and a proliferative stage **(G–O)**. **(A)** Infective zoospores seek out a suitable host cell. **(B)** Healthy host *A. pacificum*. **(C)** Early trophocyte within host cytoplasm. **(D)** Middle to late trophocyte consuming the host cytoplasmic contents. **(E)** Late trophocyte with a large nucleus. **(F)** Resting dormancy stage of the late trophocyte when host cell density was low or absent. **(G)** The bulged trophocyte wall of late trophocyte. **(H–J)** Emergence of the mature trophocyte from the trophocyte wall. **(K)** The sporocyte undergoing palintomic sporogenesis. **(L–O)** Progressively sporogenesis stages in the development of beaded sporocytes resembling a rosary.

The new parasitoid *P. dinoexitiosum* shares many morphological and developmental characteristics with other Perkinsozoan parasites (Supplementary Table 1). For example, *P. dinoexitiosum* shows an infection cycle typical of Perkinsozoan parasites, consisting of an infective stage (free-swimming zoospores), a growth stage (trophocyte), and a proliferative stage (sporocyte) (Figure 8). The similar infection cycle is also observed in Syndinean parasites such as *Amoebophrya*, although they form a vermiform before zoospore production instead of the formation of sporangium observed in Parviluciferaceae members (Coats and Bachvaroff, 2013; Jephcott et al., 2016). At the growth stage, *P. dinoexitiosum* trophocyte showed nuclear enlargement, delayed fission, and synenergide development, as seen in Parviluciferaceae members. In addition, *P. dinoexitiosum* showed several ultrastructural characteristics (e.g., apical complex structures including rhoptries, conoid-associated micronemes, micronemes, and bipartite trichocysts) commonly observed in Perkinsozoan parasites (Supplementary Table 1). All these ultrastructural characteristics were also observed in the recently described parasitoid *M*. *nigrum* (Reñé et al., 2021). Nonetheless, *P. dinoexitiosum* has remarkable morphological and developmental characteristics that distinguish it from other Perkinsozoan parasites at family or, presumably, a higher level. Morphologically, it shows the densely packed structure of trophocyte without a large vacuole or hyaline material during the growth stage, which is easily observable in Parviluciferaceae members. The most distinguishable characteristic of the new parasitoid is its sporogenesis. Among all currently known Perkinsozoan parasites, only *P. dinoexitiosum* produces sporocytes that are linked together like a string of beads by palintomic extracellular sporogenesis. Such beaded sporocytes have been commonly reported in Syndinean parasites, such as *Euduboscquella* species (Coats and Bachvaroff, 2013), but not in Perkinsozoan parasites. In addition, sporogenic division in *P. dinoexitiosum* occurs extracellularly, unlike all other Perkinsozoan parasites, in which it occurs in the sporangium or in the host cytoplasm. Taken together, these conspicuous characteristics of sporogenesis in terms of pattern and location make *P. dinoexitiosum* highly distinguishable from all other Perkinsozoan parasites formally described to date, thereby raising the need to erect a new family containing the new species.

**FIGURE 9:**
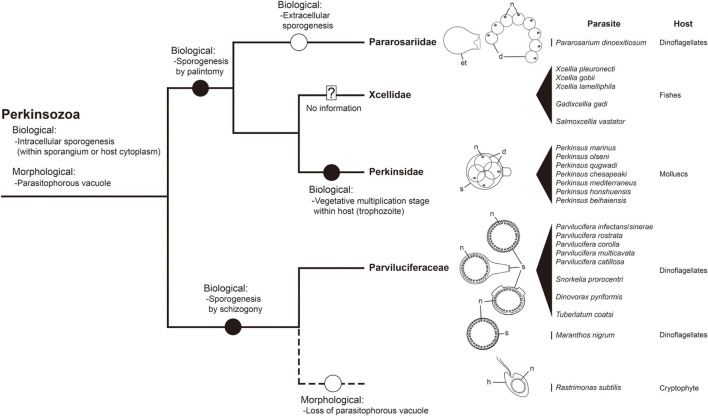
A framework for inferring biological and morphological characters evolution in the phylum Perkinsozoa. Evolution of biological and morphological characters is considered relative to the combined small and large subunit (SSU and LSU) rDNA phylogeny at the family level. Highlighted circles in branches denote positions of relevant character states as constrained by parsimony. Question mark: no detailed information on life cycle and morphology; dashed line: phylogenetic uncertainty due to unavailable sequence; et, empty trophocyte wall; d, daughter cell (sporocytes); h, host; n, nucleus; s, sporangium.

**FIGURE 10:**
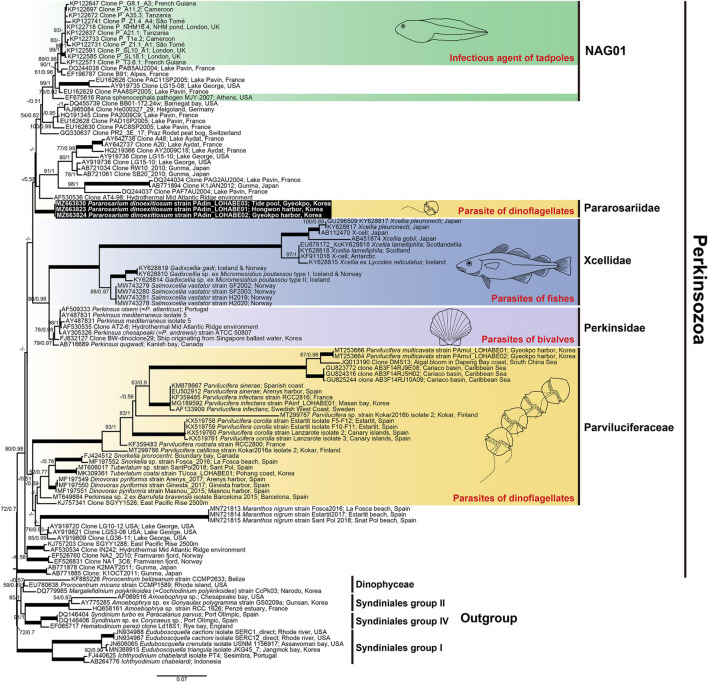
Maximum likelihood phylogenetic tree of the phylum Perkinsozoa inferred from small subunit rRNA gene sequences (1,524 bp). Sequences of the three dinoflagellates, six Syndiniales group I, three Syndiniales group II, and three Syndiniales IV were used as outgroups. The sequences of *Pararosarium dinoexitiosum* obtained in this study are indicated by the black box. The colored areas with drawings indicate the families of Perkinsozoa and their hosts. The values of bootstrap percentages **(left)** and Bayesian posterior probabilities (pp) **(right)** are shown when higher than 50% and 0.5 pp, respectively. Thick lines indicate support values of 100% and 1 pp.

The phylum Perkinsozoa was erected by Norén et al. (1999) to include *Parvilucifera infectans*, a new parasitoid infecting dinoflagellates. This group shows an apical complex, extrusomes, incomplete conoid, nucleus with non-condensed chromosomes, and anterior flagellum with unilateral row of hooks; therefore, two previous studies (Hoppenrath and Leander, 2009; Reñé et al., 2017a) attempted to infer evolutionary trends of morphological characters within the Perkinsozoa group. Unlike those morphological characters, sporogenesis of the Perkinsozoan species has received little or no attention. Although Perkinsozoan parasites share a similar life cycle consisting of an infective stage (free-swimming zoospores), a feeding and growth stage (trophocyte), and a proliferative stage (sporocyte), they show distinct differences in sporogenesis with respect to pattern and location. The ancestral Perkinsozoan parasite appears to have diverged into two groups based on characteristic patterns of sporogenesis: one group following palintomic sporogenesis (i.e., serial nuclear and cytoplasmic divisions without interruption until zoospores are formed) and the other group following sporogenesis by schizogony (i.e., multiple rounds of nuclear division followed by cytokinesis) (Figure 9). Although the group containing the family Parviluciferaceae and the genus *Rastrimonas* evolved to undergo sporogenesis by schizogony, the other group containing the three families Pararosariidae, Perkinsidae, and, probably, Xcellidae, evolved to follow palintomic sporogenesis. Remarkably, palintomic sporogenesis in the family Pararosariidae occurs extracellularly, which is contrary to intracellular sporogenesis in all Perkinsozoan parasites known to date. Further studies are need to examine why such a reversal from intracellular to extracellular sporogenesis occurred and what are its benefits from a view point of evolution to species in the family Pararosariidae. Although sporogenesis of Xcellidae members and NAG01 remains unknown, Perkinsidae members have further developed an additional characteristic stage, i.e., the vegetative multiplication stage within the host, referred to as trophozoite. By comparison, sporogenesis in Parviluciferaceae members and that in the genus *Rastrimonas* follow a division pattern by schizogony, with subsequent divisions occurring within the sporangium in the former and in the host cytoplasm without being surrounded by a parasitophorous vacuole or sporangium in the latter. Such a similar pattern in sporogenesis between the two taxa may allow us to infer that the genus *Rastrimonas* may be phylogenetically closer to the family Parviluciferaceae than to the families Pararosariidae, Xcellidae, and Perkinsidae, albeit the exact phylogenetic position of *Rastrimonas subtilis* is unknown at present because of the lack of available rDNA sequences. Given the division pattern occurring within the sporangium (Reñé et al., 2021) and phylogenetic position shown in the present study (Figure 10 and Supplementary Figure 1), the recently described parasitoid *M*. *nigrum* also appears to follow a sporogenic pattern by schizogony in the framework proposed from this study (Figure 9). This in turn indicates that *M*. *nigrum* may be not distantly related to the family Parviluciferaceae and rather be within the family. The more findings of new parasitoids and respective information on sporogenesis in the future would help improve our understanding of the evolution of biological characteristic in the phylum Perkinsozoa, as well as to better understand the phylogenetic position of *M*. *nigrum*.

In addition to the unique sporogenesis, another interesting finding from the present study is that *P. dinoexitiosum* could form a resting dormancy stage in late trophocyte when the available hosts are low or absent. The members of the family Parviluciferaceae are known to be also capable of forming the resting dormancy stage under the conditions mentioned above (Norén et al., 1999; Alacid et al., 2015). However, there is a difference in the timing of the formation of resting dormancy stage between Parviluciferaceae members and *P. dinoexitiosum*. While the Parviluciferaceae members form resting dormancy stage in late sporocyte (i.e., after replicative stage), *P. dinoexitiosum* does in late trophocyte (i.e., after feeding stage). Why the parasites display such a difference in timing of the formation of the resting dormancy stage remains at present unknown. Nonetheless, the formation of resting dormancy stage in the Perkinsozoan parasites seems to apparently provide a mechanism for survival and persistence when the conditions (e.g., host density and/or environmental parameters) are unfavorable to the parasites. The resting dormancy stage has also been commonly reported in *Perkinsus* species when their trophozoites are placed in an anoxic condition (Auzoux-Bordenave et al., 1995; Perkins, 1996). At this anoxic condition, trophozoites are transformed into hypnospores (resting stage), which are characterized as enlarged cell size and thick cell wall. The hypnospores undergo zoosporulation when they are placed in aerated seawater.

Perkinsozoa members parasitize a diverse array of host organisms, ranging from single-celled eukaryotes such as dinoflagellates and cryptophytes to mollusks and fishes (Mackin et al., 1950; Norén et al., 1999; Brugerolle, 2002; Freeman et al., 2017). Recently, tadpoles and, presumably, green algae were shown or suggested to be hosts of Perkinsozoan parasites (Davis et al., 2007; Mangot et al., 2013; Chambouvet et al., 2015; Jobard et al., 2020); however, they were not formally described. Of note, before the current study, all Perkinsozoan parasites infecting dinoflagellates were phylogenetically placed in the family Parviluciferaceae. Very recently, Reñé et al. (2021) reported a new parasitoid of the dinoflagellates, *Maranthos nigrum*, which is thought to be distantly related to the family Parviluciferaceae. Unlike their result, however, phylogenetic analyses performed in the present study showed that *M*. *nigrum* is placed at the base of the Parviluciferaceae members although the bootstrap supports are relatively low (Figure 10 and Supplementary Figure 1). By comparison, phylogenetic analysis of *P. dinoexitiosum* in the present study revealed that this species is indeed distantly related to the family Parviluciferaceae and more closely related to the families Perkinsidae and Xcellidae. This result suggests that parasites infecting dinoflagellates are much more widespread within the phylum Perkinsozoa than previously assumed. Furthermore, it is noteworthy that the wide host range of *P. dinoxeitiosum*, which includes eight dinoflagellate genera, is comparable to that of Parviluciferaceae members (Garcés et al., 2013; Lepelletier et al., 2014; Alacid et al., 2020; Rodríguez and Figueroa, 2020). Further studies are expected to unveil the widespread phylogenetic distribution of new Perkinsozoan parasitoids infecting dinoflagellates.

## Taxonomic Summary

Alveolata Cavalier-Smith, 1991

Myzozoa Cavalier-Smith and Chao, 2004

Perkinsozoa Norén et al., 1999

Perkinsea Levine, 1978

Pararosariidae fam. nov. Jeon and Park

DIAGNOSIS: Parasitic marine Perkinsea cell. Uninucleate trophocyte with non-condensed nucleus. Resting dormancy stage at late trophocyte. Extracellular sporogenesis. Reproduction by palintomy producing biflagellate zoospores.

TYPE GENUS: *Pararosarium* gen. nov. Jeon and Park

*Pararosarium* gen. nov. Jeon and Park

DIAGNOSIS: Pararoariidae endoparasitic in dinoflagellates. Fully developed trophocyte with densely packed structure, discharging a mature trophocyte giving rise to extracellular sporogenesis. Uninucleate trophocyte with rounded body, smooth surface, lacking flagella. Zoospore with bipartite trichocysts without a dense globule in the basal body.

TYPE SPECIES: *Pararosarium dinoexitiosum* sp. nov. Jeon and Park

ETYMOLOGY: Genus name *Pararosarium* is neuter and a combination from the Latin neuter nominative singular adjective *parasiticum* (parasitic) and the Latin neuter nominative singular noun *rosarium* (rosary), referring to the beaded sporocytes during sporogenesis, as observable by optical microscope.

*Pararosarium dinoexitiosum* sp. nov. Jeon and Park

DIAGNOSIS: Trophocyte variable in diameter depending on the host dinoflagellate size or on the intensity of multiple infection. Zoospore sigmoid-shaped (3 μm in length and 1.7 in width), with a rostrum, two heteromorphic flagella of different lengths, and a rounded nucleus with condensed chromatin in a reticulated pattern.

ETYMOLOGY: Species name *dinoexitiosum* is neuter and a combination of *dino*- referring to dinoflagellates and the Latin neuter nominative singular adjective *exitiosum* (deadly), referring to the infection leading to the death of the dinoflagellate host.

TYPE LOCALITY: Hongwon Harbor, Seocheon, Korea (36°09′24″N, 126°30′08″E)

HOLOTYPE: Both platinum sputter-coated stubs used for SEM and resin-embedded samples used for TEM of all life cycle stages of *P. dinoexitiosum* have been deposited at the National Marine Biodiversity Institute of Korea, Republic of Korea under the code MABIK PR00043272 to PR00043273 and MABIK PR00043274 to PR00043276, respectively.

## Data Availability Statement

The datasets presented in this study can be found in online repositories. The names of the repository/repositories and accession number(s) can be found below: GenBank; MZ663823 and MZ668307 of strain PAdin-LOHABE01; MZ663824 and MZ668305 of strain PAdin-LOHABE02; MZ663830 and MZ668306 of strain PAdin-LOHABE03.

## Author Contributions

All authors listed have made a substantial, direct, and intellectual contribution to the work, and approved it for publication.

## Conflict of Interest

The authors declare that the research was conducted in the absence of any commercial or financial relationships that could be construed as a potential conflict of interest.

## Publisher’s Note

All claims expressed in this article are solely those of the authors and do not necessarily represent those of their affiliated organizations, or those of the publisher, the editors and the reviewers. Any product that may be evaluated in this article, or claim that may be made by its manufacturer, is not guaranteed or endorsed by the publisher.

## Footnotes

1
https://blast.ncbi.nlm.nih.gov/Blast.cgi

## Supplementary Material

Supplementary Video 1 Development of *Pararosarium dinoexitiosum* in the dinoflagellate host *Alexandrium pacificum*.
