# Supplementary material for: A Novel Parasitoid of Marine Dinoflagellates, Pararosarium dinoexitiosum gen. et sp. nov. (Perkinsozoa, Alveolata), Showing Characteristic Beaded Sporocytes
Source: Front Microbiol. 2021 Nov 29;12:748092. doi: 10.3389/fmicb.2021.748092 (PMC8667275; doi:10.3389/fmicb.2021.748092)
Supplement: Supplementary file 2 [file Table_1.docx]

Table S1. Comparison of the morphological characters of *Pararosarium dinoexitiosum* with other Perkinsozoan parasites.

|  |  | ***Pararosarium dinoexitiosum*** (This study) | ***Tuberlatum coatsi***  (Jeon and Park, 2019) | ***Dinovorax pyriformis*** (Reñé et al., 2017a) | ***Snorkelia prorocentri*** (Leander and Hoppenrath, 2008) | ***Parvilucifera* spp.** (Alacid et al., 2020; Figueroa et al., 2008; Jeon and Park, 2020; Lepelletier et al., 2014; Norén et al., 1999; Reñé et al., 2017b) | ***Maranthos nigrum* (**Reñé et al., 2021) | ***Perkinsus* spp.** (Azevedo, 1989; Casas et al., 2004;  McLaughlin et al., 2000) | ***Rastrimonas subtilis*** (Brugerolle, 2002, 2003) |
| --- | --- | --- | --- | --- | --- | --- | --- | --- | --- |
| Trophocyte | Infection location | host cytoplasm | host cytoplasm | host cytoplasm | host cytoplasm | host cytoplasm | host cytoplasm | host tissues | host cytoplasm |
| /Sporocyte | Parasitophorous vacuole | Y | Y | Y | Y | Y | Y | Y | Absence |
|  | Large nucleus | Y | Y | Y | Y | Y | Y | Y |  |
|  | Large vacuole or hyaline material | N | Y | Y | Y | Y | Y | Y |  |
|  | Number of cyst wall layer | 2 | 2 | 2 | 2 | 4 | 1 | 4 |  |
|  | Cyst wall (µm) | 0.43~0.55 | 0.33 | 0.35 | 0.34 | *0.19~0.24  /0.64~0.68 | 0.11~0.15 | 1.8 ~ 3.5 |  |
|  | Cyst diameter (µm) | 21.2 ~ 24 (in *Alexandrium pacificum*) | 19 ~ 34 (in *Alexandrium catenella*) | 19.4 ~ 22? (in *Prorocentrum micans*) | 20 ~ 25 (in *Prorocentrum fukuyo*i) | 11 ~ 72 (depends on host size) | depends on host size | 20 ~ 135 |  |
|  | Cyst surface | smooth | smooth | smooth | smooth | processes | smooth | smooth |  |
|  | Sporogenesis location | extracellular | sporangium | sporangium | sporangium | sporangium | sporangium | sporangium | host cytoplasm |
|  | sporogenesis by palintomy | Y | N | N | N | N | N | Y | N |
|  | Resting dormancy stage | Y (trophocyte) | Y (sporocyte) | ? | ? | ?/Y (sporocyte in *P. infectans/sinerae*) | ? | Y (hypnospore) | Y (resting-cyst) |
| Zoospore | Zoospore shape | sigmoid | sigmoid | sigmoid | reniform | elongated/teardrop^a, b^ | sigmoid | ellipsoidal | elongated |
|  | Zoospore length (µm) | 3 ± 0.05 (n=9) | 2.8 | 6 | 4 | 4~6/2.5~2.9^a, b, c^ | 6 | 3.7 ~ 4.5 | 7 |
|  | Zoospore width (µm) | 1.6 ± 0.03 (n=9) | 1.3 | 3 | 1.5 | 1~2.2 | 3 | 2.4 ~ 2.9 | 1.5 |
|  | Anterior flagellum (µm) | 8.3 ± 0.04 (n=4) | 6.1 ± 0.1 | both flagella are similar in length | longer flagellum | 12.7~15/7^c^ | 6~7 | 12.7^e^/5.5^f^ | as long as the body, shorter anterior flagellum |
|  | Posterior flagellum (µm) | 5.4 ± 0.11 (n=8) | 4.1 ± 0.1 |  | shorter flagellum | 2.2~4.3/1.5^c^ | 6~7 | 10.7^e^/3^f^ |  |
|  | Hairs of anterior flagellum | Y | Y | Y | ? | Y | N | ?/Y^f^ | ? |
|  | Swelling of posterior flagellum | Y | Y | ? | ? | Y/?^a, b^ | N | ? | ? |
|  | Dense globule in the basal body | N | N | N | N | Y/?a, b | Y | Y | N |
|  | Heteromorphic pair of central microtubules in anterior axoneme | Y | Y | Y | Y | Y | ? | Y | ? |
|  | Refractile body | N | Y | N | Y | Y | Y | Y | N |
|  | Alveoli | Y | Y | Y | Y | Y | Y | Y | Y |
|  | Bipartite trichocysts | Y | Y | Y | Y | N | Y | N | N |
|  | Condensed genetic material in zoospore nucleus | Y | Y | Y | Y | Y | Y | Y | Y |
|  | Condensed genetic material distribution | reticulate | peripheral | ovoid | peripheral | peripheral | peripheral | peripheral | irregular |
|  | Micronemes | Y | Y | Y | Y | Y | Y | Y | Y |
|  | Reduced pseudoconoid | ? | 4 | 4 | 4~5 | 4~5/?^a^ | 7 | 20~39 | 4~5 |
|  | Conoid-associated micronemes | Y | Y | ? | Y | Y | ? | Y | Y |
|  | Rhoptries | Y | Y | Y | ? | Y | Y | Y | ? |

*Wall thickness without and with transparent layer

**References**

Alacid E, Reñé A, Gallisai R, Paloheimo A, Garcés E, Kremp A (2020) Description of two new coexisting parasitoids of blooming dinoflagellates in the Baltic sea: Parvilucifera catillosa sp. nov. and Parvilucifera sp.(Perkinsea, Alveolata). Harmful Algae 100: 101944.

Azevedo C (1989) Fine structure of Perkinsus atlanticus n. sp.(Apicomplexa, Perkinsea) parasite of the clam Ruditapes decussatus from Portugal. The Journal of parasitology: 627-635.

Brugerolle G (2002) Cryptophagus subtilis: a new parasite of cryptophytes affiliated with the Perkinsozoa lineage. European Journal of Protistology 37: 379-390.

Brugerolle G (2003) Apicomplexan parasite Cryptophagus renamed Rastrimonas gen. nov. European journal of protistology 1: 101.

Casas SM, Grau A, Reece KS, Apakupakul K, Azevedo C, Villalba A (2004) Perkinsus mediterraneus n. sp., a protistan parasite of the European flat oyster Ostrea edulis from the Balearic Islands, Mediterranean Sea. Diseases of aquatic organisms 58: 231-244.

Figueroa RI, Garcés E, Massana R, Camp J (2008) Description, host-specificity, and strain selectivity of the dinoflagellate parasite Parvilucifera sinerae sp. nov.(Perkinsozoa). Protist 159: 563-578.

Jeon BS, Park MG (2019) Tuberlatum coatsi gen. n., sp. n.(Alveolata, Perkinsozoa), a New Parasitoid with Short Germ Tubes Infecting Marine Dinoflagellates. Protist 170: 82-103.

Jeon BS, Park MG (2020) Parvilucifera multicavata sp. nov.(Alveolata, Perkinsozoa), a New Parasitoid Infecting Marine Dinoflagellates Having Abundant Apertures on the Sporangium. Protist 171: 125743.

Leander BS, Hoppenrath M (2008) Ultrastructure of a novel tube-forming, intracellular parasite of dinoflagellates: Parvilucifera prorocentri sp. nov.(Alveolata, Myzozoa). European Journal of Protistology 44: 55-70.

Lepelletier F, Karpov SA, Le Panse S, Bigeard E, Skovgaard A, Jeanthon C, Guillou L (2014) *Parvilucifera rostrata* sp. nov.(Perkinsozoa), a novel parasitoid that infects planktonic dinoflagellates. Protist 165: 31-49.

McLaughlin S, Tall B, Shaheen A, Elsayed E, Faisal M (2000) Zoosporulation of a new Perkinsus species isolated from the gills of the softshell clam Mya arenaria. Parasite 7: 115-122.

Norén F, Moestrup Ø, Rehnstam-Holm A-S (1999) Parvilucifera infectans Norén et Moestrup gen. et sp. nov.(Perkinsozoa phylum nov.): a parasitic flagellate capable of killing toxic microalgae. European journal of protistology 35: 233-254.

Reñé A, Alacid E, Ferrera I, Garcés E (2017a) Evolutionary Trends of Perkinsozoa (Alveolata) Characters Based on Observations of Two New Genera of Parasitoids of dinoflagellates, Dinovorax gen. nov. and Snorkelia gen. nov. Frontiers in Microbiology 8.

Reñé A, Alacid E, Figueroa RI, Rodríguez F, Garcés E (2017b) Life-cycle, ultrastructure, and phylogeny of Parvilucifera corolla sp. nov.(Alveolata, Perkinsozoa), a parasitoid of dinoflagellates. European journal of protistology 58: 9-25.

Reñé, A., Alacid, E., Gallisai, R., Chambouvet, A., Fernández-Valero, A.D., and Garcés, E. (2021). New Perkinsea parasitoids of dinoflagellates distantly related to Parviluciferaceae members. Frontiers in microbiology, 2199.
