# Supplementary figures and images for: A Novel Parasitoid of Marine Dinoflagellates, Pararosarium dinoexitiosum gen. et sp. nov. (Perkinsozoa, Alveolata), Showing Characteristic Beaded Sporocytes
Source: Front Microbiol. 2021 Nov 29;12:748092. doi: 10.3389/fmicb.2021.748092 (PMC8667275; doi:10.3389/fmicb.2021.748092)

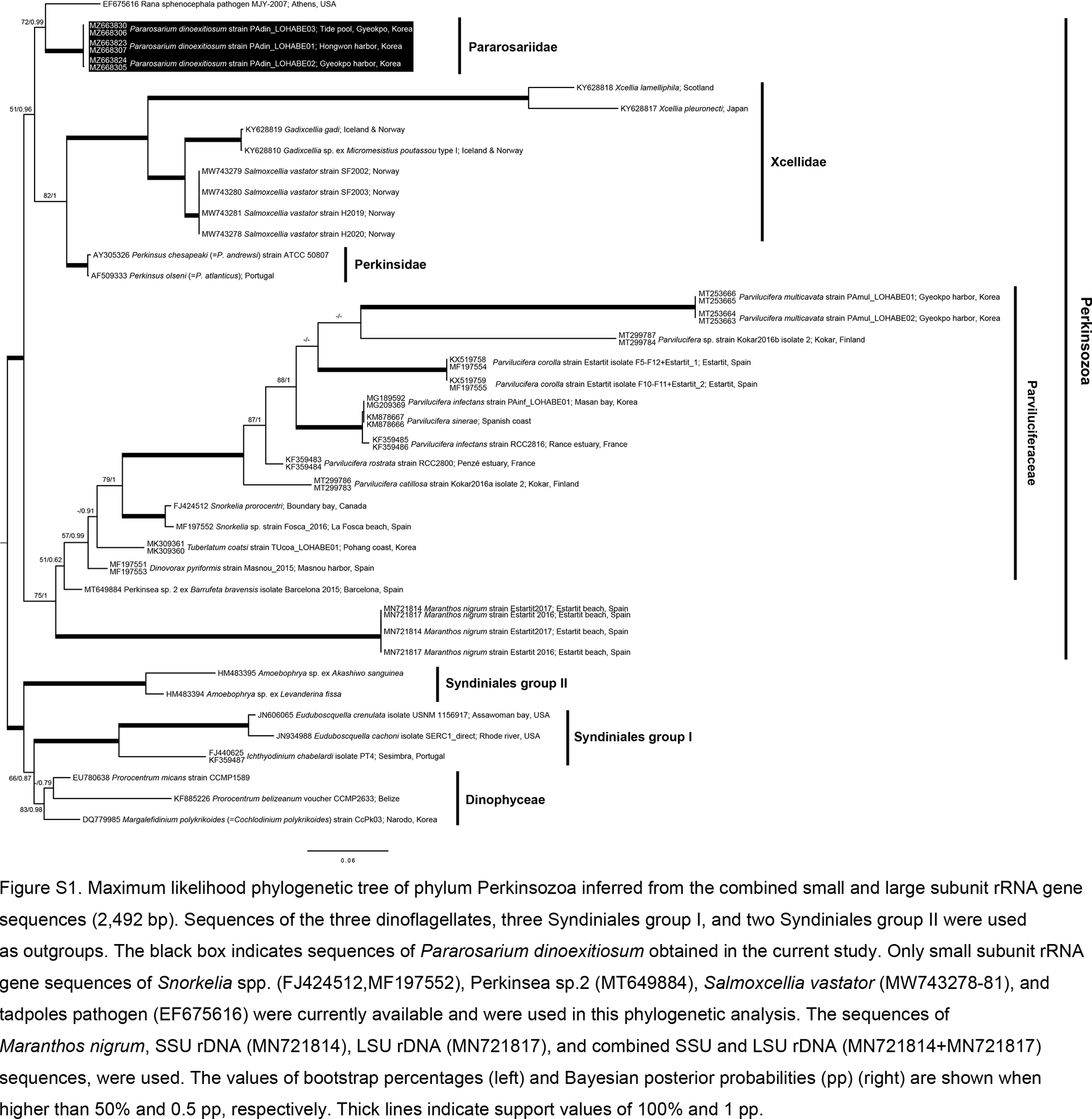

Supplement: Supplementary file 3 [file Image_1.TIF]
